# miR-8b is involved in brain and eye regeneration of *Dugesia japonica* in head regeneration

**DOI:** 10.1242/bio.058538

**Published:** 2021-06-29

**Authors:** Hongjin Liu, Qian Song, Hui Zhen, Hongkuan Deng, Bosheng Zhao, Zhonghong Cao

**Affiliations:** 1 School of Life Sciences, Shandong University of Technology, Zibo 255049, China; 2 Laboratory of Developmental and Evolutionary Biology, Shandong University of Technology, Zibo 255049, China

**Keywords:** Planarian, *miR-8b*, Brain regeneration, miRNA, Eyespot

## Abstract

MicroRNAs (miRNAs) are a class of evolutionarily conserved small non-coding RNAs that regulate gene expression at the translation level in cell growth, proliferation and differentiation. In addition, some types of miRNAs have been proven to be key modulators of both CNS development and plasticity, such as *let-7*, *miR-9* and *miR-124*. In this research, we found *miR-8b* acts as an important regulator involved in brain and eyespot regeneration in *Dugesia japonica*. *miR-8b* was highly conserved among species and was abundantly expressed in central nervous system. Here, we detected the expression dynamics of *miR-8b* by qPCR during the head regeneration of *D. japonica*. Knockdown *miR-8b* by anti-MIRs method caused severe defects of eyes and CNS. Our study revealed the evolutionary conserved role of miR-8b in the planarian regeneration process, and further provided more research ideas and available information for planarian miRNAs.

## Abstract (precis)

**Summary:** Most miRNAs in planarians are homologous to humans and other mammals, and may also play a similar regulatory role. Knockdown *miR-8b* planarian *miR-8b* induces brain and eyespot defects during head regeneration.

## INTRODUCTION

MicroRNAs (miRNAs) are short, endogenous, non-coding sequences of about 22 nucleotides in length. They modulate the expression of their target genes by mainly binding to partially complementary sites in the 3′ -UTR of the target mRNA (Li et al., 2017). Hence, miRNAs play critical role in almost all biological processes by regulating gene expression post-transcription (Sasidharan et al., 2017). Furthermore, miRNA expression is greatly regulated by multiple biological conditions, and together with transcription factors, constitutes the nodes of the genome-wide gene expression regulatory mesh network (McNeill and Van Vactor, 2012). Some relevant surveys validate that miRNAs can determine the expression of near 30% of genes and proteins, and participate in almost all physiological and pathological processes in organisms, such as cell proliferation, differentiation, apoptosis, autophagy and cell migration (Coolen and Bally-Cuif, 2009; Kim and Sung, 2017). Due to a single miRNA controlling numerous gene targets, dysregulation of miRNAs are involved in diverse pathologies, for instance in cancer (Iorio and Croce, 2017), autoimmune conditions (Zhu et al., 2013), and developmental disorders (Bian and Sun, 2011). Current surveys have shown that some miRNAs are involved in the regulation of the regeneration, neoplasm proliferation and differentiation of planarians (Krishna et al., 2019). The field of miRNA is developing rapidly, and miRNA is considered to be a key regulator of gene expression. In *Schistosoma japonicum*, *miR-8* turned out to be an outstanding marker for distinguishing sensory organs, which included eyes, antennae, and sensory organs of the pseudopodium, including neural as well as non-neural tissue (Christodoulou et al., 2010; Etebari and Asgari, 2013). In *S. japonicum*, the higher abundance of miR-8 in mature adults implies that *miR-8* had a unique role to play in stage of ripeness (Huang et al., 2009). In *Drosophila melanogaster*, *miR-8* has been proved to be a growth-inhibiting miRNA in terms of Notch signaling. Overexpressing *miR-8* in the eye suppresses the proliferation effects of Notch signaling. Notch ligand Serrate, as a direct target regulated by *miR-8*, can be blocked by *miR-8* to inhibit its cell proliferation and growth (Vallejo et al., 2011). In *D. melanogaster*, some results suggest that *miR-8* family members play an evolutionarily conserved role in regulating the Wnt signaling pathway. First, *miR-8* effectively antagonized the WG pathway at multiple levels from ligand secretion to target gene transcription. Furthermore, mammalian *miR-8* homologs promote adipogenesis in bone marrow stromal cells by inhibiting Wnt signaling. (Kennell et al., 2008). *miR-8* regulates immune homeostasis in *Drosophila* by targeting two immune genes, receptors Toll in the Toll immune pathway and transcription factors Dorsal (Lee and Hyun, 2014). The results from small RNA sequencing of *Schmidtea Mediterranea* regeneration blastemal showed that *miR-8b* highly expressed in posterior blastemal at 7 days after amputation (Sasidharan et al., 2013). The *miR-8a* is usually highly conserved in metazoans, such as it accumulates in the nervous system of sea hares (González-Estévez et al., 2009). Despite the fact that the *miR-8b* is extremely expressed in the adult brain of vertebrates, its function during neurogenesis is still unknown.

Planarian have amazing regeneration ability, they can regenerate any lost part including a functional brain in few days. Planarians have a methodical nervous system consisting of a double-lobulated brain and a couple of ventral nerve cords, which are connected by connective neurons. After being cut or damaged, planarians can regenerate the entire worm body, mainly because of the abundance of adult stem cells in its body – neoblasts (Gehrke and Srivastava, 2016). Neoblasts can be differentiated into various cells in the body of planarian, which play an influential role in the development, regeneration and self-stable maintenance of tissues and organs (Tran and Gentile, 2019). The eyes are located on the dorsal part of the ganglion of the head. The correlation between invertebrate system and vertebrate models relies in part on the presence of homology between most bilateral eyes, which is difficult to establish only from morphological studies. During planarian regeneration, the newly-formed brain and eyes were detected within 24 to 48 h post amputation. Although primordia of brain and eye differentiation was evident at 3 days post-amputation (dpa), brain function was restored only 7 days after amputation (Deochand et al., 2016). Comparative molecular genetics has been successful in establishing a degree of corporate ancestry between vertebrate and invertebrate of visual systems (Lapan and Reddien, 2012). The planarian eye is composed of pigment cells and light-energy neurons, forming goblet organs (Deochand et al., 2016). Pigment cells form a half-moon pattern at the proximal end of the transparent glass. Light-energy neurons project into the striated muscles of the goblet, and their axons form the optic chiasmus (Okamoto et al., 2005). Studies have shown that there are two types of photoreceptor neurons on both sides of planarians’ eyes, striated muscle (microvilli) dependent on conserved R-optin signaling pathway and ciliated dependent C-optin signaling pathway (Arendt, 2003). The embryonic growth of numerous eye types involves Sine oculis, Pax-6, Eyes absent and Otx gene family members (Callaerts et al., 1999; Mannini et al., 2004; Pineda et al., 2000; Umesono et al., 1999*)*. These results indicate that photoreceptor cell kinds existed in advance of the presence of bilaterally symmetrical animals and that the common ancestor of bilaterally symmetrical animals was also commonly used in existing eyes using transcription factors developed in the eye (Nilsson, 2009). We deliver anti-miR-8b into vital planarians to detect the role of *miR-8b* during head regeneration, and found knockdown *miR-8b* result in severe regeneration defects of brain and eyes. These results demonstrated a crucial function for *miR-8b* in the generation and maintenance of the brain and eyes of planarians.

## RESULTS

### Knockdown of *miR-8b* induced planarian brain and eyespot defects in the course of head regeneration

**Fig. 1.**
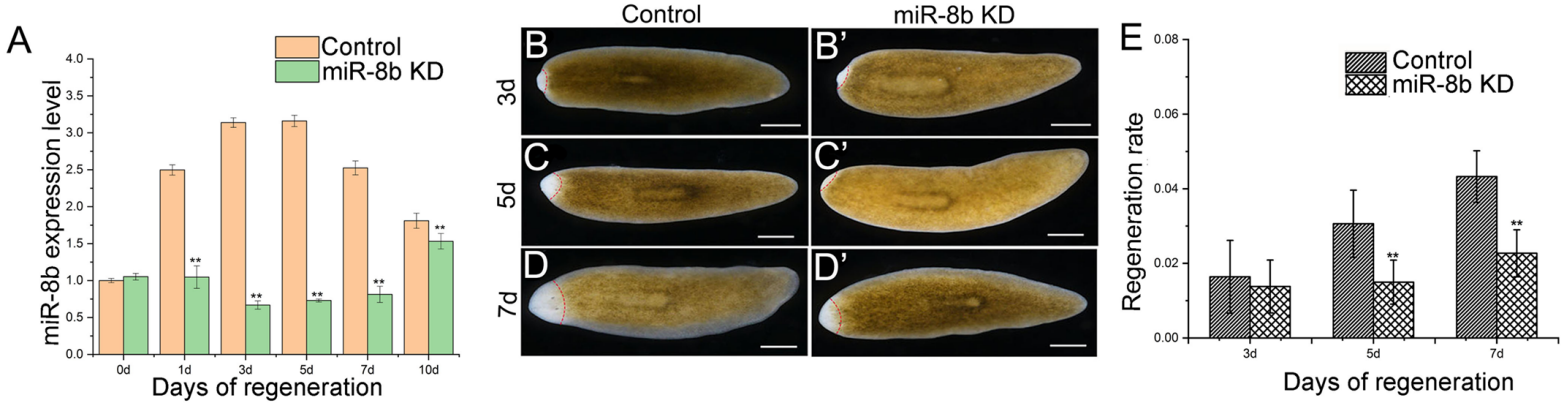
**Head regeneration of planarian from trunk fragments.** (A) The expression levels of *miR-8b* in regeneration fragments at 1 day, 3 days, 5 days, 7 days and 10 days, as determined by qPCR (*n*=3 biological replicates). Asterisks indicate statistical differences (*P<0.05; **P<0.01), pink represents the control worms, and green represents the miR-8b KD worms. (B–D) Normal regenerated brains (*n*=30 worms). (B′–D′) The phenotypes of regenerated brain in *miR-8b* KD animals at Re-3 days, 5 days, 7 days (*n*=30 worms). The dashed red lines marks the separation of the regeneration area from the rest. Scale bars: 0.5 mm. (E) The regeneration rate of planarian at Re-3 days, 5 days, 7 days. Grey-lined shading represents the control worms, and gridlines represents the miR-8b KD worms.

To investigate the expression pattern of *miR-8b* during head regeneration, we performed relative quantitative real-time PCR at different stages (Fig. 1A). The result demonstrated that *miR-8b* was significantly enhanced at the first day post amputation, and at the fifth day, the highest expression level was detected. It means that *miR-8b* may play some regulatory roles in head regeneration. In order to determine the role of *miR-8b* in the brain regeneration, we used RNA interference (RNAi) to knock out the expression of miR-8b. We soaked the amputated worms in 2.5 µM solutions of antisense Oligo nucleotide DNA of miR-8b, and detected the knockdown effects by RT-qPCR (Fig. 1A). For *miR-8b*-KD planarian, their ability of regeneration was suppressed and minor blastema tissues were produced in comparison to the control groups (Fig. 1B–D, *n*=15). In comparison to controls, from the third day of regeneration, the regeneration rate was decreased. The regeneration rate was calculated by the formula: regeneration rate=the area of regeneration/the area of the total planarian. Besides, the area of newly-formed blastemal was significantly reduced at 5 and 7 days compared to controls (Fig. 1E). These results suggest that loss function of *miR-8b* leads to reduced growth rates during brain regeneration in planarian.

**Fig. 2.**
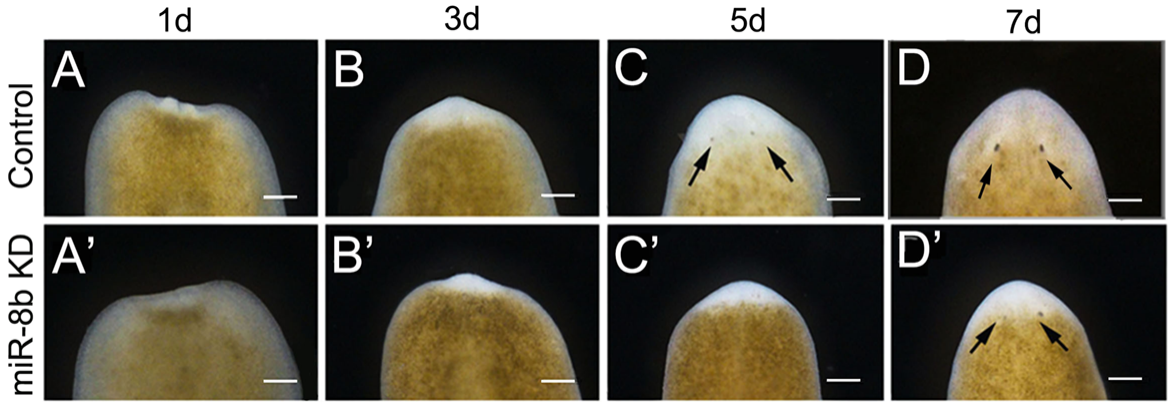
**Eyespot regeneration of *miR-8b* KD from trunk fragments.** (A–D) Normal regenerated eyespot (*n*=30 worms). (A′–D′) The phenotypes of regenerated eyespot in *miR-8b* KD animals (*n*=30 worms). Anterior is up. Scale bars: 0.5 mm.

**Fig. 3.**
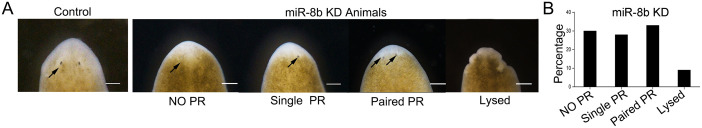
**Knockdown of *miR-8b* leads to impaired regeneration.** (A) Animals treated with 2.5 μM anti-miR-8b showed eye regeneration defects (*n*=30 worms). (B) The percentage of animals that show diverse classifications of photoreceptor (PR) blemishes observed in regenerated animals following 2.5 µM anti-miR-8b treatment (*n*=30 worms). Anterior is up. Scale bars: 0.5 mm. Control and Single PR panels are reproduced from Fig. 2D, D’, respectively.

In addition, compared to the control (Fig. 2A–D), obvious defects were detected in eyespot regions during the regeneration (Fig. 2A′–D′). The animals treated with 2.5 µM anti-miR-8b demonstrated multiple types of eye blemishes after 7 dpa, and we treated 30 worms in total, including small eyes (10/30, 33%), the complete absence of eyes (9/30, 30%), cyclopes (8/30, 28%). A few animals (3/30, 9%) displayed wounds in the head district and afterwards lysed additionally (Fig. 3A). These findings propose that *miR-8b* contributes to eyespot regeneration in planarian.

### *miR-8b* is involved in the reinstatement of brain

**Fig. 4.**
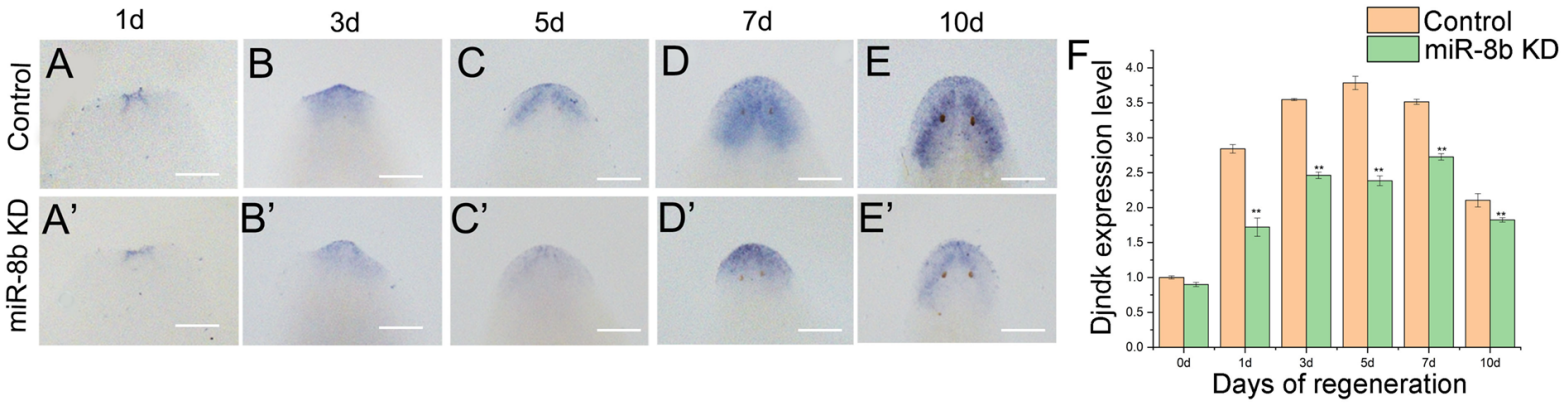
**Expression patterns of trunk fragments of *Djndk* during head regeneration.** (A–E) WISH of *Djndk* in control worms (*n*=5 worms). (A′–E′) WISH of *Djndk* in *miR-8b* KD worms (*n*=5 worms). Anterior is up. (F) Relative expression levels of *Djndk* in regeneration fragments at regeneration for 1 day, 3 days, 5 days, 7 days and 10 days, as determined by qPCR (*n*=3 biological replicates). Scale bars: 0.5 mm. Pink represents the control worms, and green represents the *miR-8b* KD worms.

With the purpose of obtain a superior comprehension of the role of *miR-8b* in the process of regeneration, control as well as *miR-8b* KD animals were reviewed on different stage animals through whole-mount *in situ* hybridization (WISH). *Nou-darake* (*ndk*) was found to be particularly expressed in the head (Cebrià et al., 2002). To conform whether *miR-8b* affects the regeneration of cranial nerve after amputation in planarian, *Djndk* were used as brain regeneration mark by WISH in decapitated planarians after *miR-8b* KD treatment (Fig. 4A–E). The results revealed that the positive signals in *miR-8b* KD regenerated planarians were considerably reduced in comparison to the control animals’ blastema at 1 and 3 dpa (Fig. 4A′,B′). Inverted U-brain was not regenerated in the newly regenerated brain area of planarian on day 5 and day 7, and the expression of *Djndk* was significantly reduced. Inverted U-shaped structure appeared at 10 days, but the positive signal was significantly reduced (Fig. 4C′–E′). In order to quantitatively evaluate the changes in gene expression of control and RNAi worms, we used qPCR to determine the relative expression levels of Djndk. Our quantitative analysis indicated that *miR-8b* KD generated a substantial diminishment in the expression level of *Djndk*, which was in agreement with our outcomes through WISH. All things considered, these findings recommend that deficiency of *miR-8b* function may disrupt brain formation during regeneration.

### *miR-8b* knockdown influence *eye53* expression level of planarian

**Fig. 5.**
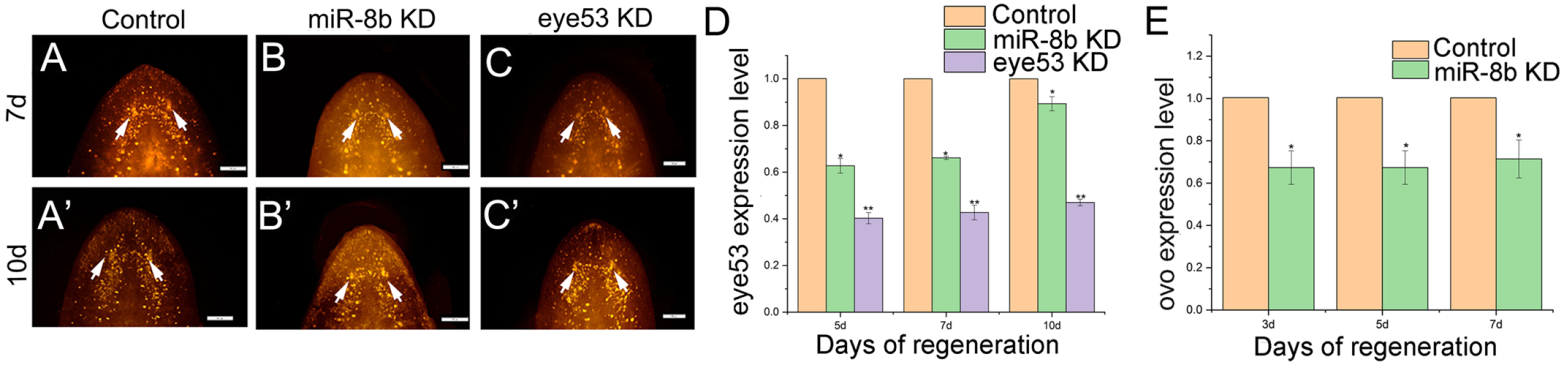
**Expression patterns of trunk fragments of *eye53* during planarian head regeneration.** (A,A′) WISH of *eye53* in control animals (*n*=5 worms). (B,B′) WISH of *eye53* in *miR-8b* KD treatment animals (*n*=5 worms). (C,C′) WISH of *eye53* in *eye53* KD treatment animals (*n*=5 worms). Anterior is up. Scale bars: 0.2 mm. (D) Relative expression levels of *eye53* in trunk fragments at Re-5 days, 7 days, and 10 days, as determined by qPCR (*n*=3 biological replicates). Asterisks indicate statistical differences (*P<0.05; **P<0.01). Pink represents the control worms, green represents the miR-8b KD worms, and purple represents eye53 KD. (E) Relative expression levels of ovo in regeneration fragments at regeneration for 3 days, 5 days, 7 days, as determined by qPCR. Asterisks indicate statistical differences (*P<0.05; **P<0.01). Pink represents the control worms, and green represents the miR-8b KD worms.

Photoreceptor cells and pigment cells are the two main characteristic cell types in the planarian eye. The light-sensing organs on the back of the head (the eyes) are made up of two types of cells: pigment cells and visual neurons (photoreceptors). Pigment cells line up into a half-moon shaped eye cup, and the visual bipolar neurons lie outside the cup. Dendrites of visual neurons are distributed within the pigment cup, forming striated muscle structures containing opsin. In previous studies, neuro-specific genes were classified into three categories according to the time of expression during brain regeneration. Among them, *eye53* started to be expressed at 5 days of regeneration and were thus categorized as late-expressing genes. *eye53* is expressed in photoreceptor neurons as well as brain cells. In our study, WISH exposed that eye53-RNAi treatment produced the obvious brain and eyespot blemishes at regenerate for 7 days and 10 days (Fig. 5). As expected, compared with the control group, the overall size of the regenerated eyes was significantly reduced in *miR-8b*-knockdown worms, indicating a decrease in the quantity of differentiated cells. We used qPCR to detect the relative expression levels of *eye53* in the control groups and RNAi trunk fragments to quantitatively assess *eye53* gene expression changes in *miR-8b*KD and eye53 RNAi animals, (Fig. 5D). This is significant that miR-8b knockdown resulting in a decrease in the expression level of *eye53*, and our quantitative analysis showed that the results were consistent with those of WISH. WISH was able to demonstrate the efficacy of *eye53* RNAi, substantial reduction in endogenous *eye53* levels and remarkably poor positive signals were discovered in eye53 RNAi planarians, which in accord with our results regard to the normal animals. We also detected down expression of *ovo* gene during head regeneration of miR-8b KD worms, the results showed as Fig. 5E. These results future implied that the *miR-8b* plays an important role in the survival and specification, differentiation of eye progenitor cells.

## DISCUSSION

This makes planarians an outstanding model organism for studying the role of miRNAs in stem cell function, thanks to a type of pluripotent stem cell called neocells, which allows planarians to regenerate all lost body tissue after amputation. Although several genes have been identified as crucial regulators for anterior destiny conclusion and regeneration of the nervous system, much still remains to be fully understood. It's essential for the modality and role of the nervous system to see how these genes coordinate in time and space to establish developmental transitions (Bonar and Petersen, 2017; Seebeck et al., 2017). The conserved physiological role of *miR-8* has been reported many times, because it is actually classified among exceedingly conserved miRNAs in organisms like nematodes through to humans (Hyun et al., 2009). Deep sequencing was performed to identify novel and regulated microRNAs in *Dugesia japonica* and *Schmidtea mediterranea* (Cao et al., 2020; Friedländer et al., 2009; Lu et al., 2009; Palakodeti et al., 2006). Our study has demonstrated enhancement for the *miR-8b* during discrete phase of homeostasis and head regeneration of planarians in the nervous system. In the newly formed brain, *miR-8b* KD animals displayed considerable insufficiency. *miR-8b* interferes with the phenotype after regeneration at 3, 5, and 7 dpa suggests that it might play a role in the patterning of visual neurons and in the organization of the brain. We investigated the expression of *Djndk* in the nervous system because a reduction in the extent of the brain should demonstrate flaws in the origination as well as sustain of neural primary cells. By interfering with the function of miR-8b in planarian regeneration, we tested this hypothesis. We were able to demonstrate that *miR-8b* KD animals show significant deficits in brain regeneration.

Planarian eyes do not contain complex optical refraction systems, such as lenses, so they can only sense the intensity of light but not image it. But, like the eyes of other higher animals, they absorb light and convert it into chemical energy, which is then converted into electrical energy to regulate their physiological activities. Studies have shown that brain malformations can lead to secondary eye defects in planarians (Cowles et al., 2013). To reveal that miR-8b KD planarians can cause defects in their brain and visual neuronal tissue, we used eye-specific markers. This finding proposes that miR-8b is concerned with brain and eye regeneration in planarians. The knockdown of miR-8b would block the regeneration of the cranial nerve and optic nerves. This investigation furnishes a useful message on the mechanism of head regeneration in planarians.

Most miRNAs in planarians are homologous to humans or other mammals, and may also play a similar gene regulatory function in planarians. Accurate regulation of miR-8b will be established in future investigations. This notwithstanding, our data provide strong evidence that miR-8b in the production and maintenance of the cerebral nervous system and in the visual system pattern of planarian regeneration has an important role to play.

## MATERIALS AND METHODS

###  Animal conditions and treatments

In this work planarians appertain to the species *D. japonica*, obtained from the Boshan, Zibo City, Shandong Province, China. The planarians were cultivation in darkness at 20°C in Lushan water and fed with fresh river shrimp once a week. The planarians were starved for 1 week prior to all experiments. Regenerated fragments were obtained by p osterior transection of the auricle. The research did not look at endangered or protected species. The collection and experiment of planarians were agreed by the Animal Protection and Use Committee of Shandong University of Technology.

### RNAi

RNAi is considered to be a key technique for gene function analysis of planarian. For miR-8b KD, planarians were amputated after auricles, and the tail fragments were immediately collected in a 24-well plate to regenerate for 10 days. Planarians were exposed to 2.5 µM antisense oligomer DNA of miR-8b in the dark at 20°C, and equal length nucleotides of random sequences were used as the control. The antisense oligomer DNA designed according to DNA and RNA hybridization rules, and antisense oligomer DNA synthetized by Sangon Biotech. Amputated animals were soaked in anti-miR-8b solution for 7 days, and exchange solutions every 48 h. The template of eye53 dsRNA was amplified using the following PCR program: 95°C for 5 min, followed by 35 cycles of 95°C for 50 s, 55°C for 50 s, 72°C for 30 s, and 72°C for 10 min. The eye53 F-primer sequence is 5′-TTTTAGTTGTTATTTGTTATCTCTGC-3′ and R-primer is 5′-TGTCATACACTCCAACGAAATA-3′. The ovo F-primer sequences is 5′-GTACTGGTACTGGAATTAGTCGATC-3′ and R-primer sequences is 5′-ATAACTTCATTAAAACTCCTCAAAC-3′. Consequently, eye53 and ovo primer sequences were synthesized were synthesized according to the manufacturer's specification *in vitro* (MEGAscript™ RNAi Kit). Double stranded RNA (dsRNA) was synthesized *in vitro* and the standard 2.5 µM dsRNA were immersed in the worms. Control animals were immersed with water. The regeneration rate of planarian head is perceived by calculating the area of the blastemal at different regeneration stages. WISH and qPCR validated the effectiveness of RNAi.

### WISH

The planarians were cut by posterior transection of auricle and the fragments were left to regenerate over 1, 3, 5, 7 and 10 days. WISH was conducted as reported previously (King and Newmark, 2013). The worms were primary immersed in phosphate buffer solution containing Nacetyl-cysteine solution treated for 5 min, and then immobilized in paraformaldehyde treated with 15 min. In order to improve the display of the signal expression, worms were decolorized in 6% H_2_O_2_ (H_2_O_2_ in methanol) for 6 h. In brief, the digoxigenin (DIG)-labeled antisense RNA probes were designed according to the Roche labeling kit *in vitro*. The antisense RNA probe was hybridized with 1 ng/μl antisense RNA probe for 16 h at 56°C in hybrid solution. A mixture solution containing maleic acid and 10% horse serum to blocking planarian. It was then labeled with a Cy3 chromogenic substrate solution (Roche) of color chromogenic agent.

### qPCR and RNA extraction

Quantitative expression of *miR-8b* and related genes was monitored using qPCR. Total RNA was extracted from planarians with Trizol according to the manufacturer’s instructions at different time points (0, 1, 3, 5, 7, and 10 days). Washed RNA was dissolved in 20 μl nuclease-free water and the concentration was determined by the NANODROP 2000C system (Thermo Fisher Scientific). A 15–20 bp poly(A) tail was added to all RNA using Poly(A) polymerase (NovoBiotec, PAP5 104H) followed by first strand synthesis (Roche, 04379012001) using an oligo(dT) linked random primer adapter. The control treated fragments and perturbing treated fragments were detected at different time after amputation by qPCR. *miR-8b* F-primer sequence is: 5′-TAATACTGTCAGGTAAGAAT-3′, R-primer is 5′-GCGAGCACAGAATTAATACGACTC-3′, *β-actin* F-primer sequence is: 5′-ACACCGTACCAATCTATG-3′ and R-primer: 5′-GTGAAACTGTAACCTCG-3′, *Djndk* F-primer sequence is: 5′-TCACAAACTCCACCGCAGTACTTT-3′ and R-primer: 5′-GGTATGGATTAGCATTATTGAATTGTG-3′. cDNA was synthesized using a Revert Aid First Strand cDNA Synthesis Kit (Thermo Fisher Scientific). qRT-PCR was performed using SYBR Green PCR Master Mix (Roche) and internal controls were used as the *β-actin* gene. Relative RNA abundance was repeated three times and analyzed using the Roche LightCycler 48Ⅱwith the comparative Ct method (2−△△ct).

### Statistical analysis

Measurement data in the experimental data were expressed as mean±s.d. and one-way analysis of variance (ANOVA) was used for comparison between multiple groups. For all analyses, *P<0.05, **P<0.01, were considered to be statistically significant.
